# Editorial: Mechanisms of Traditional Medicinal Plants Used to Control Type 2 Diabetes or Metabolic Syndrome

**DOI:** 10.3389/fphar.2020.617018

**Published:** 2021-01-14

**Authors:** Mohamed Eddouks, Adolfo Andrade-Cetto, Michael Heinrich, Vincenzo De Feo, William C. Cho

**Affiliations:** ^1^ Team of Ethnopharmacology and Pharmacognosy, Faculty of Sciences and Technology, Moulay Ismail University of Meknes, Errachidia, Morocco; ^2^ Laboratorio de Etnofarmacología, Facultad de Ciencias, Universidad Nacional Autónoma de México, Mexico City, Mexico; ^3^ Centre for Pharmacognosy and Phytotherapy, University College London School of Pharmacy, London, United Kingdom; ^4^ Department of Pharmacy, University of Salerno, Fisciano, Italy; ^5^ Department of Clinical Oncology, Queen Elizabeth Hospital, Kowloon, Hong Kong

**Keywords:** mechanisms of action, medicinal plants, metabolic syndrome, diabetes, Treatment

**Editorial on the Research Topic**

**Mechanisms of Traditional Medicinal Plants Used to Control Type 2 Diabetes or Metabolic Syndrome**

Type 2 diabetes mellitus (T2DM), the prevalent form of diabetes, a heterogeneous, multifactorial disorder related to diminished insulin secretion, insulin resistance and related factors, such as obesity, sedentary lifestyle, stress and aging. The International Diabetes Federation estimates that without any intervention to slow down the rise in T2DM, there will be at least 700 million people with diabetes by 2045. The changes in lifestyle (lack of exercise, junk food) and increase in life expectancy, human facing more challenges from metabolic disorders (Nazarian-Samani et al., 2018; Xiao and Luo, 2018). Among the metabolic diseases, we have the cluster of conditions (hypertension, hyperglycemia, abdominal obesity, and elevated cholesterol or triglyceride levels) that when occur together are called; metabolic syndrome and T2DM (Saklayen, 2018). Medicinal plants are widely used throughout the world especially in the developing countries to manage the metabolic syndrome and T2DM (Chukwuma et al., 2019). The pathophysiological conditions engage metabolic pathways involve hundreds of enzymes, proteins, co-factors; various cells, tissues and organs especially pancreas, liver, gut, muscle, adipose tissue and kidney. It is important to understand the mechanisms underlying the medicinal plants in controlling the metabolic syndrome and T2DM (Demmers et al., 2017; Ota and Ulrih, 2017; Salehi et al., 2019). However, conceptual and methodological challenges exist. Thus, this Research Topic aims to aid to our understanding of the hypoglycemic actions by medicinal plants and derived phytochemicals. Among 27 submitted manuscripts for this Research Topic, only seven manuscripts have been accepted.

Mao et al. studied the combined use of *Astragalus* polysaccharide (AP) and berberine (BBR) on insulin resistance in IR-HepG2 Cells. Their study demonstrated that AP-BBR attenuated IR in this cell model with a concomitant reduction of H_2_O_2_ content. The study has also shown that the protein expression of p-FoxO1Ser256 and PEPCK in IR-HepG2 cells was increased while the protein expression of FoxO1 and GLUT2 was decreased. Interestingly, AP-BBR combination was able to reverse the above protein expression changes suggesting that AP-BBR attenuates IR in IR-HepG2 cells probably via the regulation of the gluconeogenesis signaling pathway. While Mata-Torres et al. focused on the study of hepatic glucose output inhibitory activity of some Mexican plants in streptozotocin-induced diabetic rat or *in vitro* assessing their inhibitory effect on glucose-6-phosphatase. The plant extracts of *Ageratina petiolaris*, *Bromelia karatas*, *Equisetum myriochaetum*, *Rhizophora mangle*, and *Smilax moranensis* inhibit hepatic glucose output. Besides, Belwal et al. updated the uses of berberine; its phytopharmacology, clinical use, its prevention and treatment of diabetes and other metabolic diseases. Berberine exhibited beneficial effect in animal models of chronic diseases, however, the authors stated that its efficacy in clinical setting has not yet been demonstrated.

In fact, berberine is an isoquinoline alkaloid from *Coptis chinensis* Franch has attracted notable attention for its pharmacological properties. Wang et al. assessed the effect of berberine on special learning memory impairment caused by diabetes. To investigate spatial learning and memory, the authors employed the Morris water maze assay in diabetic rats and the results demonstrated that berberine ameliorated spatial learning memory impairment by attenuation of β-amyloid formation through the activation of the cholinergic anti-inflammatory and insulin signaling pathways in diabetic rats. Through a systematic review and meta-analysis, Wang et al. tried to highlight the capacity of Astragaloside IV (AS-IV), a saponin isolated from *Astragalus membranaceus* (Fisch.) Bunge, which is known for beneficial pharmacological activities to treat renal diseases. Several preclinical studies have demonstrated the renoprotective effect of AS-IV through its antifibrotic, antioxidant, and antiapoptotic actions, resulting in alleviation of endoplasmic reticulum stress, inhibition of mitochondrial fission, and increased autophagic activity. On the other hand, Heydarpour et al. reviewed the pathogenesis of diabetes and concluded that some phytochemicals and plant extracts may act as alternatives to current drugs in diabetes treatment based on their modulation of the autophagy and/or TGF-β signaling pathways.

Park et al. investigated the effect of Taeumjowi-tang (TJT), a traditional Korean sasang remedy on obesity-atopic dermatitis comorbidity. The authors used a high-fat diet (HFD) and 1-fluoro-2,4-dinitrobenzene (DNFB) as obesity and Alzheimer’s disease (AD) model, they demonstrated that TJT treatment improved the symptoms related to obesity and AD especially epidermal thickness and eosinophil/mast cell infiltration, reduction in immunoglobulin E, interleukin (IL)-4, IL-6, and tumor necrosis factor-alpha (TNF-α). TJT was shown to suppress HFD/DNFB-associated inflammation-related nuclear factor-kappa beta (NF-κB) and mitogen activated protein kinase. Furthermore, treatment of TJT down-regulated the hypoxia inducible factor-1 alpha protein in the mice model of obesity-atopic dermatitis comorbidity. They suggested that TJT is a potential candidate which could be used to improve clinical symptoms related to obesity-AD comorbidity.

It is valuable to explore the beneficial effects of these plant derived medicines on the metabolic syndrome. We know this may be just the tip of an iceberg, other mechanisms of actions, cellular and molecular action sites remain to be discovered. We are convinced that those promising pre-clinical studies warrant further clinical trials.

## Author Contributions

All authors listed have made a substantial, and intellectual contribution to the work and approved it for publication.

## Conflict of Interest

The authors declare that the research was conducted in the absence of any commercial or financial relationships that could be construed as a potential conflict of interest.
